# Nickel ion release and surface analyses on instrument fragments fractured beyond the apex: a laboratory investigation

**DOI:** 10.1186/s12903-023-03434-9

**Published:** 2023-09-30

**Authors:** Sıdıka Mine Toker, Ekim Onur Orhan, Arzu Beklen

**Affiliations:** 1 Metallurgical and Materials Engineering Department, Eskisehir Osmangazi University, Eskisehir, 26040 Turkey; 2 Department of Endodontics, Faculty of Dentistry, Eskisehir Osmangazi University, Eskisehir, 26040 Turkey; 3 Department of Periodontology, Faculty of Dentistry, Eskisehir Osmangazi University, Eskisehir, 26040 Turkey; 4 Translational Immunology Research Program (TRIMM), Research Program Unit (RPU), University of Helsinki, Helsinki, Finland

**Keywords:** Nickel-Titanium Instruments, Root Canal Shaping, Instrument failure complication, Simulated body fluid, Nickel ion release

## Abstract

**Background:**

To analyse the changes in surface and nickel ion release characteristics of fractured root canal shaping instruments in a simulated body fluid environment.

**Methods:**

A total of 54 new instruments were studied. The instrument groups consisted of five different NiTi alloys and a stainless-steel alloy. To standardize instrument fracture, a torsional type of failure was created on each instrument. The fractured specimens of each instrument group were randomly divided into three static immersion subgroups of 1 h, 7-day, and 30-day (n = 3). Simulated body fluid (SBF) was prepared to mimic human blood plasma by Kokubo&Takadama protocol for *ex situ* static immersions at 37ºC. The surfaces were examined *via* scanning electron microscopy coupled with energy-dispersive X-ray spectroscopy. To determine the quantitative ion release, the retrieved SBFs were analyzed using inductively coupled plasma mass spectrometry. Two-way ANOVA and Tukey post hoc tests sought the statistical significance of the nickel ion values(p < 0.05).

**Results:**

In 1 h of immersion, the newly formed structures, exhibiting mostly oxygen signals, were widespread and evident on NiTi surfaces. In contrast, fewer structures were detected on the SS surface in that subgroup. In 7 days of immersion, a tendency for a decrease in the density of the new structures was revealed in NiTi groups. The oxygen signals on NiTi group surfaces significantly increased, contrary to their decrease in SS. Signals of sodium, chlorine, and calcium were detected, indicating salt precipitates in groups. In 30 days of immersion, salt precipitates continued to form. The Ni-ion release values in all instrument groups presented significant differences in comparison to the SBF control in all immersion periods(p < 0.001). No significant differences were observed in immersion time periods or instrument groups(p > 0.05).

**Conclusions:**

Within the limitations of the presented study, it was concluded that the fractured SS and NiTi root canal instruments release Ni ions in contact with body fluid. However, the Ni ion release values determined during the observation periods are lower than the critical toxic or allergic thresholds defined for the human body. This was due to the ionic dissolution cycle reaching a stable state from 1-hour to 30-day exposure to the body fluid of fractured instruments.

## Background

Biofilm infections caused by bacteria can induce apical periodontitis [1]. Elimination of biofilm during the cleaning and shaping phase of endodontic treatment is vital for clinical success [1]. However, the complex anatomy of canals, such as severe curvatures, irregular cross-sections, isthmuses, and fins, presents a formidable challenge for this cleaning step [2]. The cutting geometry of conventional nickel-titanium (NiTi) rotary instruments does not always match the root canal anatomy [3]. Therefore, mechanical instrumentation of complex root canals using conventional rotary systems may remove excessive dentine or leave certain areas untouched [4]. Niti instruments with various designs and concepts have been established in recent years to meet the complex anatomy of root canals.

Rotate instrument (VDW GmbH, Munich, Germany) is manufactured by Blue NiTi alloy [5]. The titanium oxide layer formed by the specific post-machining heat treatment gives the alloy its blue hue [6]. The heating procedure NiTi wire technology enables the instrument to reach the martensitic phase, making it more fatigue-resistant than instruments that are predominantly in the austenitic phase, which is more unyielding [7]. Similar to Hyflex CM instruments (Coltene-Whaledent, Allstetten, Switzerland), OneCurve (MicroMega SA, Besancon Cedex, France) is manufactured from NiTi alloy using a proprietary heat treatment with a controlled memory property that makes the instruments more resistant to fatigue [8]. The ProTaper Next system (Dentsply Maillefer, Ballaigues, Switzerland) possesses an innovative asymmetric characteristic that allows only two cutting edges to contact the canal wall during continuous rotation [9]. This system comprises austenitic NiTi M-Wire alloy and possesses better mechanical characteristics compared to conventional NiTi alloy [9]. The miniScope instrument (ScopeEndo, Yozgat, Turkey) was recently introduced. According to its manufacturer, MS is produced through a thermal post-grinding process. The instrument has a triangular design and reduced threading in its active portion [10].

Instrument separation is a sudden complication that occurs when the plastic limits of the instrument are exceeded in the process of root canal shaping [11]. The residual fragment is not always bypassed or removed in all cases. The presence of leftover instrument fragments can impede the effectiveness of orthograde cleaning and disinfection methods, hence potentially enhancing the survival rate of biofilms [12–14]. In addition, management of these complications is relatively more complex than that of the middle or coronal one-third: coronal access is very limited, and accordingly, removing, negotiating, or bypassing the fractured tip is a challenge. If the separated tip is located at the apical third, when it cannot be removed or bypassed, “leaving in situ” can be accomplished with periodic follow-ups [15–19]. Solomonov [18] reported that removing the fragment should not be attempted. This approach has been reported as advantageous when the separated tip is small or positioned in the apical part of the canal or beyond the apex and the handling of retrieval systems, even with ultrasonic vibration, is difficult [15–19]. In the case of post-treatment endodontic disease development, apical microsurgery has been recommended [18].

Broken instruments are not a reason for developing a periapical disease; some studies demonstrated cases of patients who had fractured instrument fragments beyond their root apices with no periapical inflammation or symptoms [17, 18]. Biomedical applications of SS alloy were reported to have superior corrosion resistance with lower carbon content, which is useful for preventing carbon-chromium precipitate formation [20]. Yet, the surface or ionic interactions of endodontic instruments fractured beyond the apex and surrounded by a body fluidic environment have not been specifically demonstrated; therefore, this indicates a gap in the literature.

In the past decade, much research has focused on oxide layers on metal surfaces and different ion release rates during body fluid exposures due to corrosive environments [21, 22]. And, depending on the patient’s characteristics and the amount of ion release at certain exposure periods, allergic or toxic reactions may occur [23, 24]. Therefore, to see the corrosion behaviour of alloys used in a specific biomedical application, it is crucial to monitor their corrosion and ion release tendencies in body fluid environments. Yet, it remains unclear about the corrosion patterns and potential ion release of the fractured endodontic instruments, especially during prolonged exposures to bodily fluids.

Therefore, a clinical question arises if a fractured instrument is subjected to the corrosive body fluid environment for an uncertain amount of time, creating a potential for corrosion of the implanted fragment and ion release into the body. An ‘ion release characterization model’ was recently demonstrated in NiTi root canal instruments exposed to a 0.9% saline environment [25]. Alike, metal ion release of the different types of NiTi alloys used in orthodontic appliances has been studied using a 0.9% saline model [26]. But, to the authors’ knowledge, no literature-based data is available concerning the corrosion and ion release of fractured metal instruments using a simulated body fluid or artificial blood environment representing beyond the apex.

- This study aims to clarify the effect of prolonged exposure of fractured instruments beyond the apex to corrosive body environments. More specifically, the purpose of this study was to analyse the surface and nickel ion release characteristics of fractured root canal shaping instruments in a simulated body fluid environment. The null hypothesis of the study was that there was no difference between the corrosion and ion release behaviour of fractured endodontic instruments exposed to a simulated body fluid representing beyond the apex.

## Methods

**Fig. 1:**
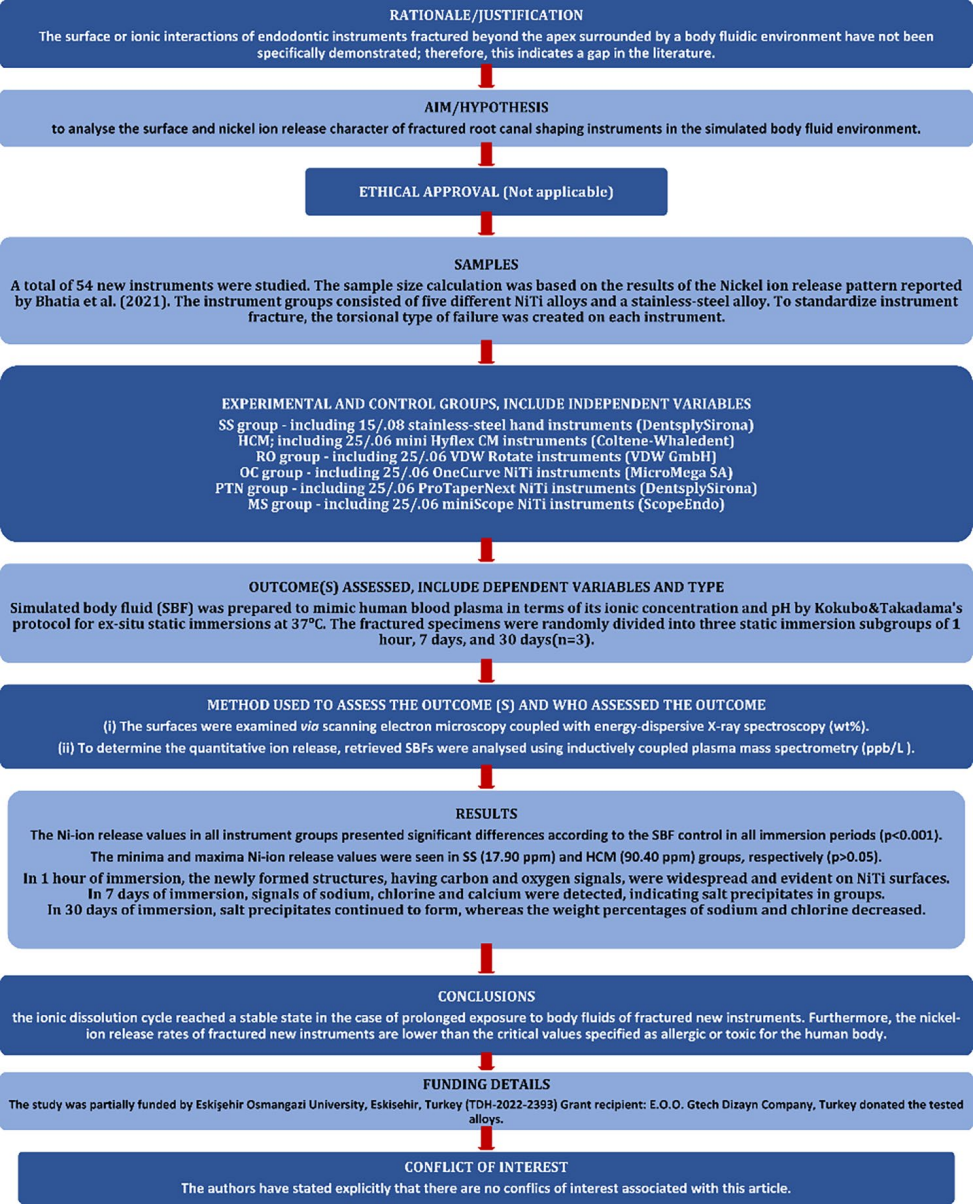
PRILE 2021 flowchart

In this study, systematic ex-situ experiments were designed to simulate in vitro conditions to obtain qualitative and quantitative datasets. The manuscript of this laboratory investigation was written according to ‘Preferred Reporting Items for Laboratory Studies in Endodontology 2021 Guidelines’ [27]. The design of the study and the results are shown in a flowchart Fig. 1.

### Root canal instruments

The sample size calculation was based on the results of the Nickel ion release pattern reported by Bhatia et al. [25]. Accordingly, a total of 54 new instruments were studied. The information about the root canal instruments is listed in Table 1. The instrument groups consisted of five different NiTi alloys (25/0.06 sizes) and a stainless-steel alloy (15/0.08 size). To make it easy to follow, the abbreviations of the instrument groups are coded in the text as follows: SS; SS hand instrument (15/0.08 C + Ready.Steel; Lot# 1367753, Dentsply Sirona, Maillefer Instruments Holding Sarl, Ballaigues, Switzerland), MS; mini SCOPE (25/0.06 Lot# GN121; Scope Endodontic System, Gtech Dizayn Co., Yozgat, Turkey), RO; Rotate (25/0.06 Lot# 291814; VDW GmbH, Munich, Germany), HCM; Hyflex CM (25/0.06 Lot# F60212; Coltene-Whaledent, Altstatten, Switzerland), OC; One Curve (25/0.06 Lot# 96,344,628; MicroMega SA, Besançon Cedex, France), and PTN; ProTaper Next (25/.06v Lot# 1593764; Dentsply-Sirona, Ballegiues, Switzerland).

**Table 1:** The information about the root canal instruments

Instrument (Manufacturer details)	Abbreviation	Size. Colour. Alloy ^Ψ^	Lot#
C + Ready·Steel hand instruments (Dentsply Sirona, Maillefer Instruments Holding Sarl, Ballaigues, Switzerland)	SS	15/0.08. Grey. Stainless steel: 69.5% Iron (CAS#13097-37-1); 18% Chromium (CAS#7440-47-3); 9% Nickel (CAS#7440-02-0); 2% Manganese (CAS#7439-96-5); 0.75% Cobalt (CAS#7440-48-4).	1367753
miniSCOPE M3 (Scope Endodontic System, Gtech Dizayn Co., Yozgat, Turkey)	MS	25/ 0.06. Yellowish. Heat-treated yellowish NiTi alloy. Ni:Ti = 55.1:44.9 (wt%/wt%).	GN121
Rotate (VDW GmbH, Munich, Germany)	RO	25/ 0.06. Blue wire: heat-treated NiTi alloy. Ni:Ti = 54.8:45.2 (wt%/wt%).	291814
Hyflex CM (Coltene-Whaledent, Allstetten, Switzerland)	HCM	25/ 0.06. Yellowish. CM wire: heat-treated NiTi alloy. Ni:Ti = 54.4:45.6 (wt%/wt%).	F83368
One Curve (MicroMega SA, Besançon Cedex, France)	OC	25/ 0.06. Yellowish. C wire: heat-treated NiTi alloy. Ni:Ti = 53.8:46.2 (wt%/wt%).	96344628
ProTaper Next X2 (Dentsply-Sirona, Ballaigues, Switzerland)	PTN	25/ 0.06v*. Grey. M-wire NiTi alloy. Ni:Ti = 55.0:45.0 (wt%/wt%).	1593764

The group assignments are arbitrarily ordered in the table. *v: variable taper. ^**Ψ**^ The information is derived from manufacturer data

All new instruments were examined under a stereomicroscope (Stemi 508; Carl Zeiss Microscopy GmbH, Göttingen, Germany) at x12.5 magnification for any signs of visible deformation. In the event that any deformation was detected, it was discarded from the study.

### Sample preparation

To standardize instrument fracture, the torsional type of failure was created on each instrument using the universal twisting apparatus (Calibration date 30.11.2022; Gtech Dizayn Co., Yozgat, Turkey) according to the International Organization for Standardization 3630–1:2008 specifications [28]. The shank part of each instrument specimen was removed, and its unmilled core part was attached to the chuck of the apparatus and tightened. The tip of the specimens was aligned at 3 mm level into a couple of brass jaws fixed to a gear motor of the apparatus and screwed. The instruments were twisted with a torque of a maximum of 35 N·m at 2 rpm constant speed until the fracturing time according to the suggestions by international standards. The data on maximum torque and angular deflection until fracture were excluded due to being outside of the scope of the aims of the study. The length of each fractured tip was measured and individually stored in 2 mL closed tubes until immersion experiments. Prior to immersion experiments, the samples were cleaned with ethanol in an ultrasound bath for 5 min. Then the surface area of each fractured sample was calculated using the truncated cone calculation. The flute depths of instruments were not considered in the calculations, as previously described [28].

### Simulation of the physiological environment

The fractured specimens of each instrument group were randomly divided into three static immersion subgroups based on the determined immersion frequencies of 1-hour, 7-day, and 30-day (n = 3).

Simulated body fluid (SBF) was prepared freshly for each experiment, which simulates human blood plasma in terms of its ionic concentration and pH. The ionic content of the SBF solution that was formulated according to the previously described protocol by Kokubo & Takadama [29] is provided in Table 2. The pH of the SBF was set at 7.4, and the pH measurements were frequently checked during the experiments. The instrument sample surface area and SBF volume ratio were set at 1:10, as described by [29]. The immersion periods were 1 h, 7 days and 30 days, during which the specimens were kept at a constant temperature of 37 ºC in order to simulate the physiological environment by an electronically controlled water bath (BM 302; Nüve A.S., Ankara, Turkey). The SBF solutions were kept still until each immersion period is completed, in order to be able to fully observe the effect of each determined immersion duration on ion release.

**Table 2:** Chemical content of the simulated body fluidic used in the static immersion experiments given in the order of addition during preparation. (M: molar)

Adding order	Chemical	Amount (g/L)
1	NaCl	7.996
2	NaHCO_3_	0.350
3	KCl	0.224
4	K_2_HO_4_.3H_2_0	0.228
5	MgCl_2_.6H_2_O	0.305
6	1 M-HCl	40 mL
7	CaCl_2_	0.278
8	Na_2_SO_4_	0.071
9	(CH_2_OH)_3_CNH_2_	6.057

### Data collection with surface analysis

Following each immersion period, the samples were retrieved from the static immersion solutions, rinsed with distilled water, and dried under the air flow of the fume hood prior to the surface examinations. Dried samples were attached to the metal stubs with an adhesive carbon tape, and topographies were examined using field-emitted scanning electron microscopy (FE-SEM) coupled with energy-dispersive X-ray spectroscopy (EDX) instruments (FE-SEM, Hitachi Regulus 8230, Hitachi High-Tech Co., Tokyo, Japan, and Ultim Extreme EDX instrument, Oxford Instruments, High Wycombe, UK). The coating process was not performed on the samples since they are metallic biomaterials.

Spectroscopic and topographic image data were collected at 150X, 300X, and 1000X magnifications. The examinations were concentrated on significant and newly detected structures formed during immersion. Following the immersion, newly formed structures or sites observed during FE-SEM imaging were chemically analyzed with EDX spectroscopy for characterization. In the EDX analyses, the chosen sites or structures were examined in detail in both point and area spectra at an accelerating voltage of 10 kV and a 10 mm working distance.

### Data collection with ion-releasing analysis

To investigate the ion release from the instrument fragments, retrieved immersion solutions were analyzed with inductively coupled plasma mass spectrometry (ICP-MS, Thermo Fisher Scientific, iCAP RQ, MA, USA) instrument. Among the constituent elements of the tested alloys, nickel, iron, copper, chromium, and molybdenum were considered for ICP-MS analyses.

Plastic tubes filled with retrieved liquid samples were kept at 37 ºC throughout the experiments. For sampling in the ICP-MS analyses, 1 mL of each retrieved SBF test solution was diluted with 1 mL HNO_3_ (65%; Merck KGaA, Darmstadt, Germany) and 1 mL H_2_O_2_ in ultrapure water for 24 h. Ultrapure water (Elga Purelab Flex 4, Raptor Supplies Limited, London, UK) was the negative control in the ion releasing analyses.

### Data analysis

The statistical evaluation concerning the Ni-ion release comparing different times and instrument groups was performed using the quantitative dataset obtained by ICP-MS analysis. Shapiro-Wilk’s normality test revealed the data were normally distributed. Two-way ANOVA and Tukey post hoc tests were performed to determine significance (p = 0.05).

## Results

### Results of surface analysis

**Fig. 2:**
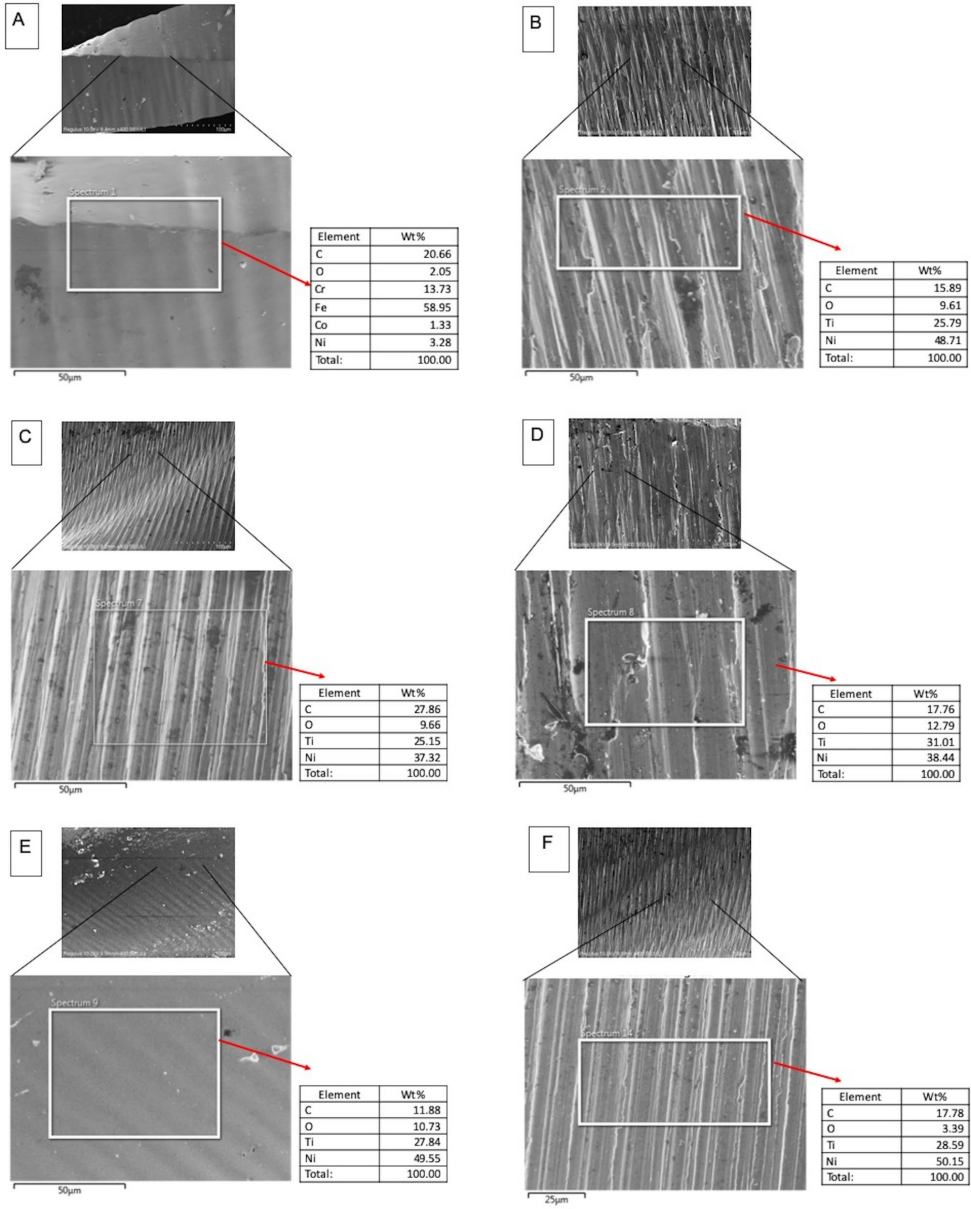
EDX spectra of the representative areas of control (with corresponding SEM micrographs) with no immersion. A; Stainless-steel hand instrument, B; miniSCOPE NiTi instrument, C; Rotate NiTi instrument, D; HyflexCM NiTi instrument, E; OneCurve NiTi instrument, F; ProTaperNext NiTi instrument. NiTi: nickel-titanium

**Fig. 3:**
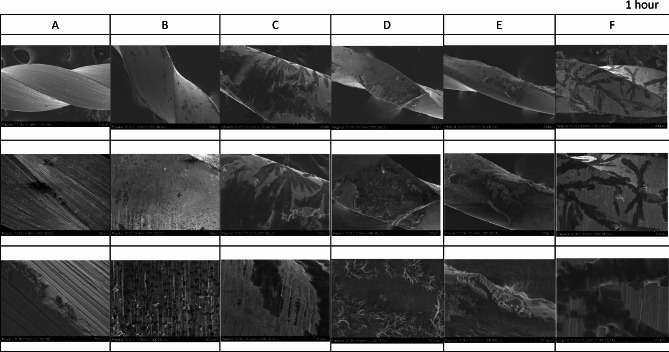
The representative scanning electron micrographs of the groups following 1-hour immersion in SBF. A; Stainless-steel hand instrument, B; miniSCOPE NiTi instrument, C; Rotate NiTi instrument, D; HyflexCM NiTi instrument, E; OneCurve NiTi instrument, F; ProTaperNext NiTi instrument. NiTi: nickel-titanium

**Fig. 4:**
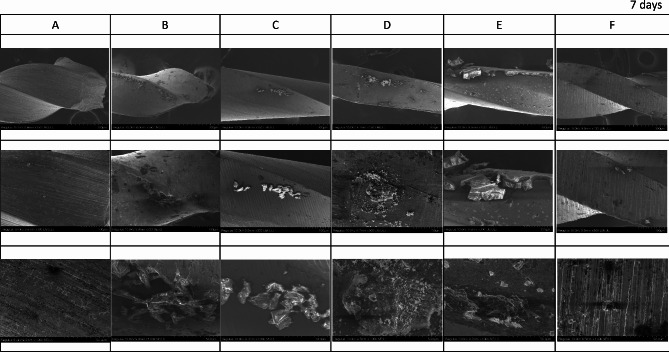
The representative scanning electron micrographs of the groups following 7-day immersion in SBF. A; Stainless-steel hand instrument, B; miniSCOPE NiTi instrument, C; Rotate NiTi instrument, D; HyflexCM NiTi instrument, E; OneCurve NiTi instrument, F; ProTaperNext NiTi instrument. NiTi: nickel-titanium

**Fig. 5:**
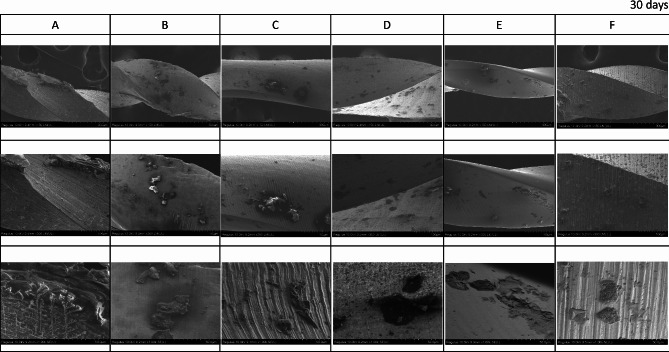
The representative scanning electron micrographs of the groups following 30-day immersion in SBF. A; Stainless-steel hand instrument, B; miniSCOPE NiTi instrument, C; Rotate NiTi instrument, D; HyflexCM NiTi instrument, E; OneCurve NiTi instrument, F; ProTaperNext NiTi instrument. NiTi: nickel-titanium

**Fig. 6:**
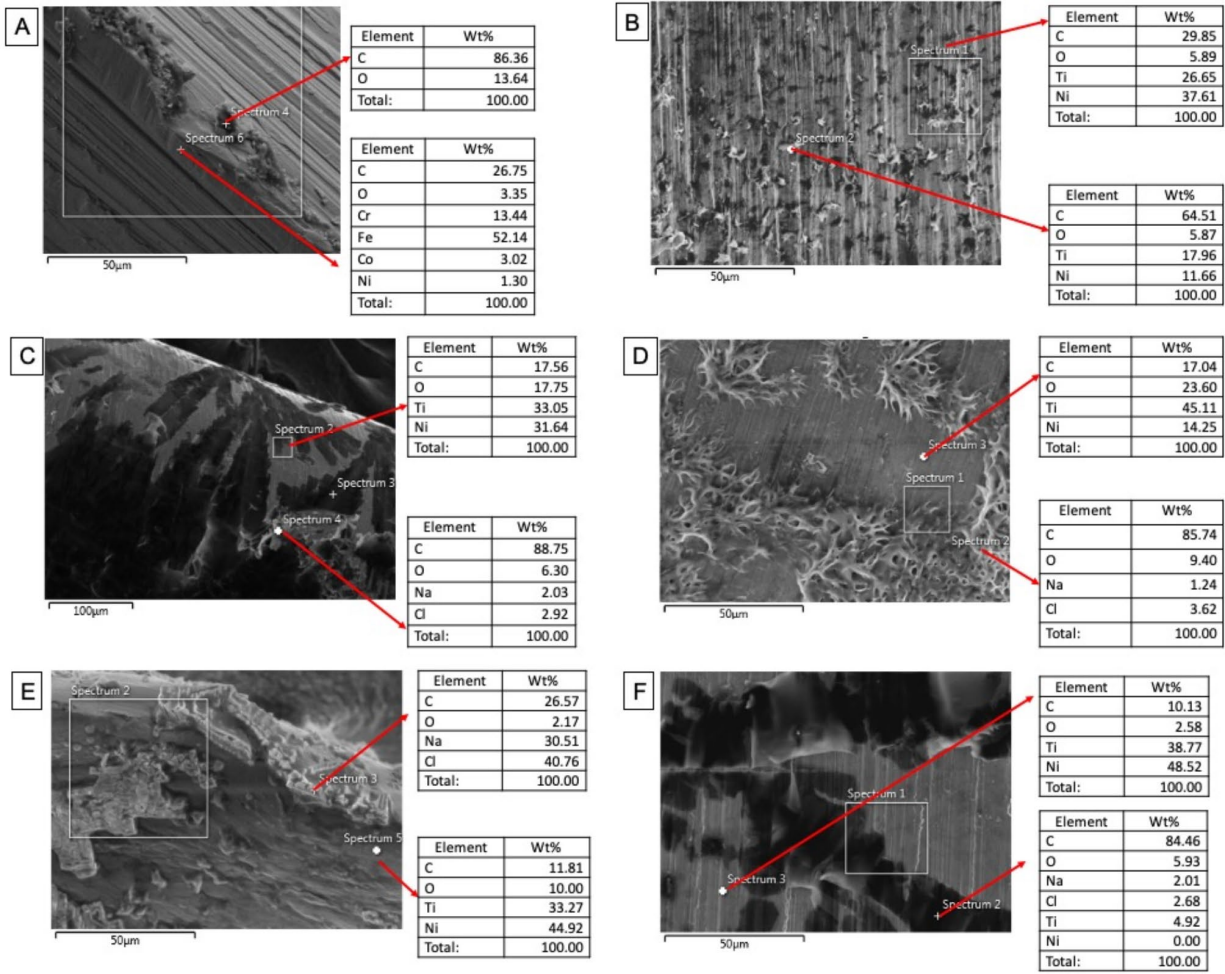
EDX spectra of the representative areas of the groups (with corresponding SEM micrographs) following 1-hour immersion in SBF. A; Stainless-steel hand instrument, B; miniSCOPE NiTi instrument, C; Rotate NiTi instrument, D; HyflexCM NiTi instrument, E; OneCurve NiTi instrument, F; ProTaperNext NiTi instrument. NiTi: nickel-titanium

**Fig. 7:**
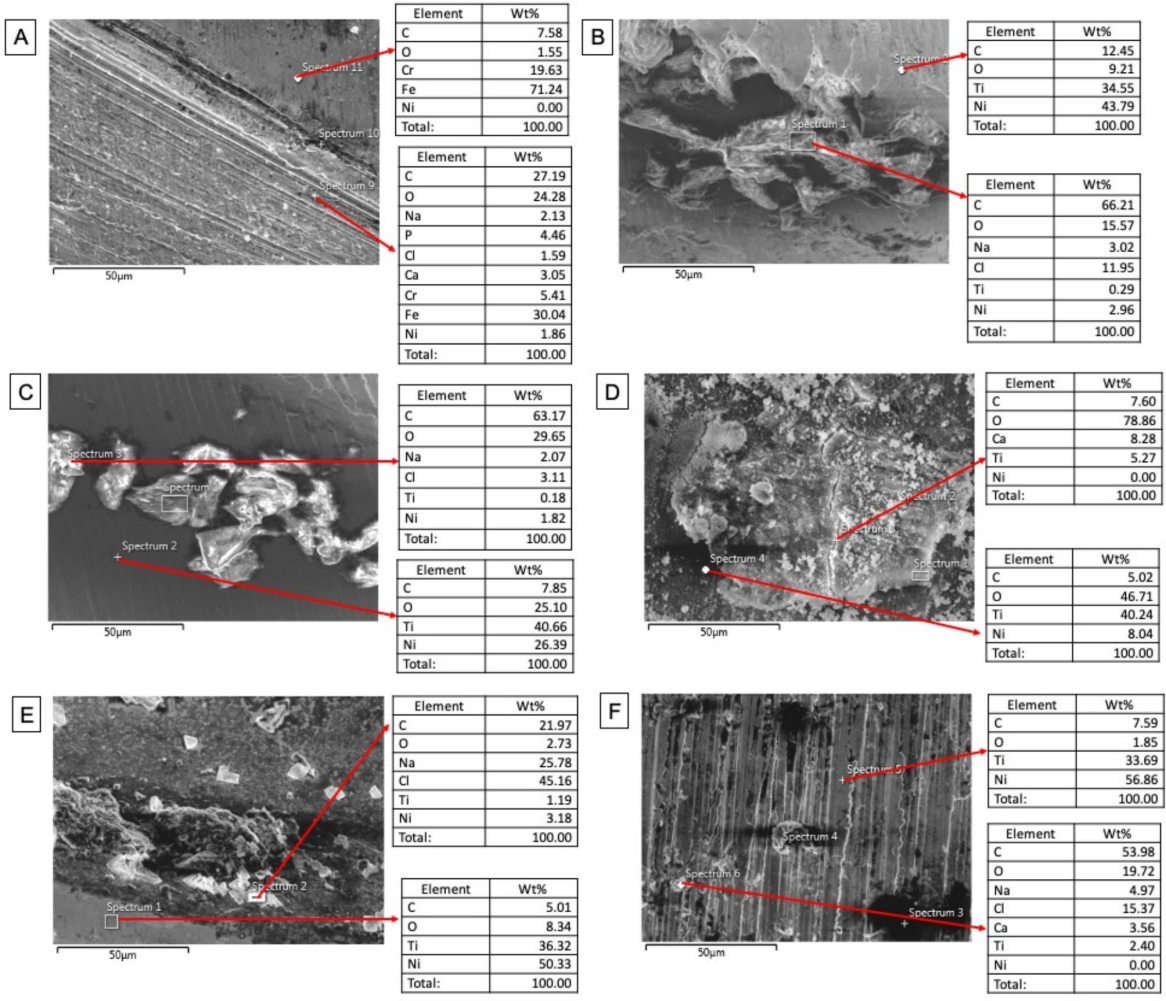
EDX spectra of the representative areas of the groups (with corresponding SEM micrographs) following 7-day immersion in SBF. A; Stainless-steel hand instrument, B; miniSCOPE NiTi instrument, C; Rotate NiTi instrument, D; HyflexCM NiTi instrument, E; OneCurve NiTi instrument, F; ProTaperNext NiTi instrument. NiTi: nickel-titanium

**Fig. 8:**
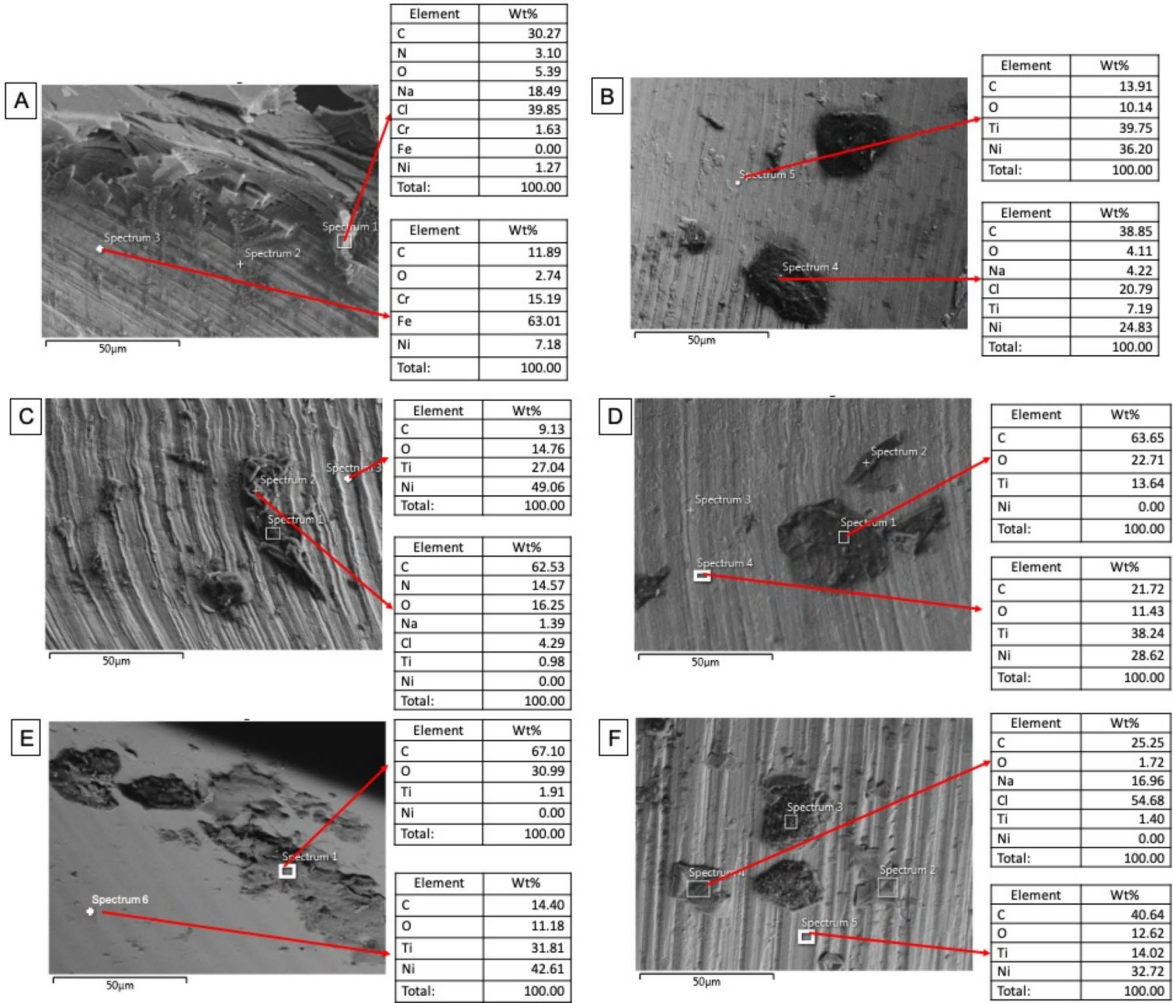
EDX spectra of the representative areas of the groups (with corresponding SEM micrographs) following 30-day immersion in SBF. A; Stainless-steel hand instrument, B; miniSCOPE NiTi instrument, C; Rotate NiTi instrument, D; HyflexCM NiTi instrument, E; OneCurve NiTi instrument, F; ProTaperNext NiTi instrument. NiTi: nickel-titanium

The FE-SEM micrographs with the EDX spectra of the control samples (non-immersed samples) are shown in Fig. 2. The FE-SEM micrographs of the representative areas of the samples following 1-hour, 7-day and 30-day of immersion in SBF are shown in Figs. 3, 4 and 5. The EDX spectra of the samples following 1-hour, 7 days and 30 days of immersion in SBF are presented in Figs. 6, 7 and 8.

### Results of 1-hour immersion

The first significant observation was the formation of new structures in all NiTi groups following the first immersion period Fig. 3. The new structures were seen as fewer and relatively homogeneously distributed on the surfaces in the MS group. The new structures spread as a “leaf-like” form in RO and PTN groups. On the other hand, significantly fewer new structures were observed on the SS.

To make a comparison, the newly formed structures were identified *via* EDX spectra from both a representative area of the observed structures and the structure-free or regular sites on the surfaces Fig. 6. In the structure-free zone, the measurements yielded mainly Ni and Ti (the primary constituent elements of the alloy itself), with signals of carbon and oxygen in the NiTi groups. The newly formed structures were remarkably rich in carbon and oxygen. In addition, the new structures showed sodium and chlorine signals in the OC group.

### Results of 7-day immersion

The overall view of the NiTi samples demonstrated a tendency for a decrease in the density of new structures Fig. 4. Especially on samples RO and PTN, the “leaf-like” spread particles were no longer evident after 7 days of immersion. Samples SS, HCM and OC exhibited smaller yet more homogeneously distributed structures in comparison to 1-hour immersion results. In the NiTi groups, relatively larger particles were observed after 7 days of immersion at higher magnifications. On the contrary, structures on the SS surface were smaller and more homogeneously distributed at higher magnifications.

The EDX results of the structures observed on surfaces following 7 days of immersion are shown in Fig. 7. Spectra obtained from the newly formed structures yielded signals of carbon and oxygen, similar to those observed after 1-hour immersion. However, significant signals of sodium, chlorine, and calcium were seen in the new structures that formed during the 7-day immersion, which can be indicators of salt precipitates that might have formed due to the interaction with the SBF environment.

EDX spectra obtained from the structure-free areas yielded signals of the constituent elements of the NiTi alloys, with additional signals of carbon and oxygen, for all of the samples, which were similar to the observations from the 1-hour immersion. The oxygen percentage on NiTi groups was significantly higher after 7 days of immersion as compared to the values observed following 1-hour immersion while the oxygen percentage on the SS surface on day 7 was lower than that after 1-hour immersion.

### Results of 30-day immersion

The overall views of the NiTi groups were similar to those observed after 7 days of immersion, with arbitrarily distributed new structures on surfaces Fig. 5. On the SS surface, however, larger, bulkier and randomly distributed structures were also evident, in addition to the previously observed homogeneous and smaller precipitates. At higher magnifications, on NiTi groups, the smaller and more homogeneously distributed particles become more apparent.

EDX analysis of the new structures formed following 30 days of immersion in SBF revealed that they were mainly rich in carbon and oxygen, similar to the observations from 1-hour and 7-day immersions. Signals of sodium and chlorine, indicating the formation of salt precipitates, were also observed only in the SS, RO, and OC groups after 30 days of immersion. On the other hand, the weight percentages of sodium and chlorine were lower than the observed amounts after 7 days of immersion. In the structure-free areas, Ni, Ti, carbon, and oxygen signals were detected. In terms of the weight percentages of carbon and oxygen, the trend of increase or decrease in comparison to the previous immersion periods varied for each group Fig. 8.

### Results of ion-releasing analysis

The detected Ni-ion release amounts (parts per billion per liter, ppb/L) in the immersion solutions are shown in Table 3. The Ni-ion release values in all instrument groups presented significant differences according to the SBF control in all immersion periods (p < 0.001). No significant differences were observed in immersion time periods or instrument groups (p > 0.05). The minima and maxima of Ni-ion release values were seen in the SS (17.90 ppm) and HCM (90.40 ppm) groups, respectively (p > 0.05).

**Table 3:** Mean quantitative Nickel ion release values (and standard deviations) to the simulated body fluidic environment in the static immersion experiments

#	Groups	1-hour (ppb/L)	7-day (ppb/L)	30-day (ppb/L)
Control	SBF	7.41 (7.90)*	7.15 (3.68)*	8.86 (3.49)*
1	SS	35.02 (9.01)	29.43 (10.10)	32.48 (4.06)
2	MS	51.22 (10.33)	33.76 (3.13)	34.56 (3.30)
3	RO	35.53 (3.68)	34.10 (3.60)	33.40 (2.28)
4	HCM	68.10 (29.48)	54.43 (30.73)	34.53 (0.38)
5	OC	37.24 (3.48)	28.80 (6.51)	29.97 (2.10)
6	PTN	41.20 (3.39)	46.00 (17.14)	59.12 (44.90)

* Two-way ANOVA followed by Tukey post hoc tests presented statistical significance (p < 0.001). ppb: parts-per-billion. 1 ppb/L = 0.001 milligrams per litre. The group abbreviations are SBF: simulated body fluid, SS; Stainless-steel hand instrument, MS; miniSCOPE NiTi instrument, RO; Rotate NiTi instrument, HCM; HyflexCM NiTi instrument, OC; OneCurve NiTi instrument, PTN; ProTaperNext NiTi instrument. NiTi: nickel-titanium

The observed Ni ion release was highest following the first hour of immersion, which tended to decrease following 7 days of immersion in NiTi groups. The only group that exhibited higher ion release during the 7-day immersion period as compared to the 1-hour immersion period was the SS. In 30 days of immersion, the Ni release amounts were very similar to those measured on the 7th day of immersion for all groups, with only minor changes. Specifically, a slight decrease was observed in Ni release for SS, RO, and PTN; a slight increase was detected for MS and OC groups.

Findings of the ICP-MS results also revealed that the detected ion release amounts for the elements Fe, Cu, Cr, and Mo were very low for all groups at all immersion periods, and no significant changes were observed between immersion periods; therefore, data was not shown.

## Discussion

The ion release and surface analyses of the fractured endodontic instrument fragments were demonstrated using a valid tissue fluid scenario for the first time in the literature in the present study. The designed methodology simulated leaving the separated fragment in situ approach. Microcirculation is very limited inside the root canal. Thus, a fractured object inside the root canal lumen has limited contact with tissue fluids. Undoubtedly, microcirculation beyond the apex causes more interaction with embedded objects. Therefore, in our simulations, we preferred that the worst-case scenario ex-situ test design namely “instrument fracture at beyond the apex”. We specifically simulated the conditions where the fractured instrument is subjected to the ionic content of blood, at its original pH level and at the body temperature environment, for different time spans with a maximum of 30 days in our worst-case scenario.

The obtained results of the experiments yielded important differences and findings regarding new structure formation behaviour on specimen surfaces and ion release tendency of the alloys as a function of time and specimen surface; therefore, the null hypothesis was rejected. For the tested NiTi instruments, the first hour of SBF exposure was observed to result in more significant changes in terms of new structure formation, as compared to the tested SS instrument. The findings of this study clearly show that the manufacturing of NiTi instruments in terms of alloy properties, surface coatings, and biocompatibility is relatively more complex in comparison to the SS instruments, as described previously in studies [20, 23, 30]. In this study, the NiTi alloy groups were designated due to their individual metallurgical disparities. Specifically, the NiTi alloy groups B-E are known to be in martensitic phase, while the NiTi alloy group F is in austenitic phase. Moreover, although details about proprietary manufacturing methods are rarely disclosed, it is known that the NiTi groups are all manufactured by milling techniques. The milling process, can naturally cause surface imperfections and irregularities such as grooves, cracks, or pits [31–36]. Also, these imperfections and irregularities represent higher energy regions on the surfaces and are therefore expected to be preferential sites for the nucleation and precipitation of new structures [22, 37]. Furthermore, it is known that martensite phase can be more easily deformed under stress [38], which may result in the formation of a higher amount of surface irregularities and therefore increased roughness on the sample surface of the alloys with martensitic phase (groups B-E in the current study).

These irregularities seen on the NiTi instrument surfaces resulting from milling processing could cause the alloy surface to be more prone to precipitate formation than SS in the immersions, especially for the NiTi alloy groups B-E owing to their crystal structure and thus deformability [38]. On the other hand, SS hand instruments are manufactured using relatively fewer milling machine techniques than NiTi instruments. Accordingly, the significantly lower amount of new structure formation in the SS group is correlated with the relatively smooth and low-energy characteristics of their surfaces.

The time-dependent surface analyses indicated an increase in oxide layer thickness and the presence of some salt-like precipitates on the surfaces from 1-hour to 7-day exposure to SBF in NiTi groups. It is known that during extended exposures to bodily fluids, the protective oxide layer on surfaces of biomedical alloys goes into a continuous cycle of dissolution and reformation [21]. Also, the observed carbon-rich leaf-like structure formation during early exposure to NiTi groups was in agreement with a previous study [39]. In addition, the detected oxygen and carbon may stem from the protective coating on the NiTi surfaces, as well as a non-localized oxide layer or carbon-rich precipitate formation during the immersion procedure [22]. Based on the findings, it can be interpreted that the initially formed carbon-rich particles on the NiTi instrument surfaces were dissolved during further exposure to SBF, warranting the formation of more homogeneous and stable oxide layers on the NiTi surfaces.

The surface observations at the 30-day immersion exhibited similar views of the surfaces to those observed at the 7-day immersion in NiTi groups. This is another indication that the oxide layers formed on the surfaces have reached a stable state during the prolonged exposure to SBF. On the other hand, calcium, phosphorus, sodium, or chlorine-rich salt-like precipitates continued to form on the surfaces, especially at higher energy sites, evidencing that the dynamic dissolution-reformation processes continued as exposure to SBF proceeded.

For the SS surfaces, although the same dynamics apply, the observations exhibited the dissolution of the already existing oxide layer during the 7-day immersion period, which was followed by its reformation as well as salt-like precipitate formation up to the 30-day immersion on SBF. This finding indicates that the formation-dissolution cycle of the oxide layer during exposure to SBF takes place at a slower rate on SS than in the NiTi groups. The late initiation of the oxide layer formation-dissolution cycle followed by a slower propagation of the process in SS instruments could be explained by their relatively lower surface energy than NiTi instruments.

The initial ion release rates from the different instruments measured following one hour of immersion can be correlated to their existing surface properties resulting from manufacturing. Specifically, the Ni release rates from the instruments with higher amounts of surface irregularities, as evident in Fig. 2, especially for alloy groups B and D, are relatively higher as compared to other groups. This finding is an indication of how higher energy sites, such as stress concentration points resulting from the manufacturing process on the alloy surface can induce higher ion release when subjected to a corrosive medium [22–25]. On the other hand, following initial ion release, the oxide layer formation-dissolution cycle becomes more effective for ion release.

Regarding the relationship between oxide layer stability and ion release, our findings indicated a correlation between these two variables such that, for the NiTi groups, the ion release rates exhibited a tendency to decrease as the SBF immersion periods extended, during which the oxide layer formation process was observed to reach stability. The reason oxide layer stability on the root canal instrument surfaces is critical is the protective effect of the oxide layer against ion release, as previously reported in the studies [20–22].

The EDX findings of NiTi groups, where oxygen percentages were significantly higher following 7 days of immersion as compared to 1 h, were also in correlation with the ICP-MS results. The decrease in nickel ion release from 1-hour to 7-day immersion for NiTi groups could be explained by the increase in oxide layer thickness, which acts as a more protective coating against ion release. On the other hand, it was also observed that as the immersion period was prolonged, salt-like precipitates formed on the alloy surfaces, in addition to the oxide layer. The authors interpreted that the salt-like precipitates may also affect the ion release by blocking the micro-cracks formed on the thicker oxide layers, which are potential sites for ion release.

Ion release into the human blood medium may cause toxic or allergic reactions, which may endanger the patients’ health. For metallic implant materials, various complications may develop due to an increase in metal ion concentration in human serum. Such complications may be encountered due to the release of ions such as Ni, Al, V, and Mn. Specifically, in the case of potential Ni release, toxicity symptoms may vary from mild responses such as acute headache or nausea to very severe effects such as chronic cardiovascular, respiratory, or kidney diseases [40]. Therefore, it is essential to ensure the ion release levels remain below critical rates, especially for patients with Ni allergy or Ni hypersensitivity [25]. In the current findings, the nickel ion-releasing levels were lower than the reported critical toxic or allergenic value of 120 µg/L for human blood tissue [41]. Overall, since the Ni ion release amounts measured on the 7th and 30th days of immersion overlapped each other, it can be postulated that the oxide layers and salt-like precipitates might reach a stable state following 30 days of immersion. In other words, it was assumed that the nickel-ion release rate might not significantly increase from this point in the fractured NiTi instruments beyond the apex. Yet, the authors noted that the oxide layer dissolution and reformation cycle may trigger diffusion within the NiTi alloy, promoting the formation of different Ni-Ti phases; which may result in unsteady Ni release rates over time [37].

Similarly, as the above-mentioned process occurred at a slower rate in the SS group, decrease in ion release rate was observed during prolonged periods of immersion. A decrease in oxygen level observed in 1-hour and 7-day immersions indicated the dissolution of the existing oxide layer in SS. The increased ion release for SS from 1 h to 7 days could be explained by the dissolution of the protective oxide layer over time, which results in an increase in ion release. In contrast, the observed lower ion release rate at 30 days of immersion indicated the stability achieved at the later observation periods for SS instruments. The ICP-MS findings revealed that the fractured SS and NiTi root canal instruments released Ni ions in contact with body fluid. The minimum Ni-ion release value was detected in SS. This could be explained by the low weight% of Ni in the steel alloy. The standardized tests were made to simulate fracture; however, the longest fractured tips were seen in HCM. This could explain why HCM presented the maximum ion release value.

When a shaping instrument is inadvertently operated beyond the root apex, there is an instantaneous occurrence of local hemorrhage due to local traumatic injury. This bleeding results from physical stress to the capillary vessels in the peripheral vascular connective tissues. During the period of wound healing, there is a temporary presence of an inflammatory environment that contains fluid, which supports the processes involved in the repair process. This environment typically persists for approximately 3–4 weeks [42]. A substance that possesses the capability to facilitate the formation of apatite on its surface in simulated bodily fluid (SBF) also exhibits the ability to generate apatite on its surface within the human body. Consequently, it establishes a connection with living bone by means of this apatite layer. The aforementioned association remains valid under the condition that the item in question does not possess any constituents that elicit harmful or antibody responses. Several materials have the ability to make direct bonds with living bone, without exhibiting observable apatite development on their surfaces. Notwithstanding this constraint, the analysis of apatite development on the surface of a substance in SBF has significance in forecasting the material’s in vivo bone bioactivity, encompassing both qualitative and quantitative aspects. This methodology can be employed to evaluate bone bioactive compounds prior to doing animal tests, resulting in a significant reduction in the number of animals utilized and the duration of animal research. Consequently, this approach facilitates the expedited creation of novel bioactive materials [29]. The fractured instrument fragment beyond the apex may stay in situ for much longer than one month with no symptoms. The preferred 30-day maximum immersion period in this study was associated with the initial fluidic wound-healing process. While this environment was designed to mimic human blood plasma in terms of its ionic concentration and pH, it may not fully replicate the complex conditions present in the human body. This was considered a limitation of the in vitro nature of the present study design thereof. However, the simulated body fluidic environment is a more relevant design as compared with the physiological saline environment reported in a recent study [25]. Therefore, the findings of this study may reflect the ion release behavior of fractured instruments in vivo better than the findings of Bhatia et al. [25]. Specific rotational kinematic-related forces of the engine-driven NiTi instruments may cause them to engage beyond the apical foramen, which leads to the extrusion of debris, and the traumatic damage of the apex or periapex [43–45]. Aside from nickel-related toxicity, the overall host defense against exogenous materials depends on the degree of trauma, foreign body reactions, and the presence or absence of infectious irritants [41]. The experimental design of this study cannot simulate the inflammatory or biological outcomes of the fractured instruments. Therefore, the non-simulated inflammatory environment was considered a limitation of the present study by the authors.

Spagnuolo et al. [46] reported that sterilization cycles could cause corrosion-type surface alterations on reused shaping instruments. Further studies should be conducted to understand the effects of sterilization cycles on the ion-releasing or surface characteristics of fractured instruments at time-dependent immersions in simulated body fluids. Torque occurs due to the stress imposed on both the instrument and the tooth when operating an engine-driven shaping instrument. Hence, a monotonic torsional failure-related model was designed in order to establish a standardized failure mechanism. Consistent with the previous study’s findings, variations in ion-releasing behavior may arise due to manufacturing faults, metallurgical characteristics, and distinct types of torsional failure [47]. Additional research is required to examine the potential correlation between the torsional mode of failure and potential instrument defects or the discharge of ions.

## Conclusions

Within the limitations of the presented study, it was concluded that the fractured SS and NiTi root canal instruments release Ni ions in contact with body fluid. The ionic dissolution cycle reached a stable state from 1-hour to 30-day exposure to the body fluid of fractured instruments. It was suggested that, as a result of this behavior, Ni ion release values determined during the observation periods remained lower than the critical toxic or allergic thresholds defined for the human body. Nevertheless, this preferred study model is practical for the comparison of the ion-releasing and surface analysis of fractured instruments beyond the apex in terms of reproducibility, standardization, and visualization.

## Data Availability

The datasets used and/or analysed during the current study are available from the corresponding author on reasonable request.

## List of abbreviations

EDX: Energy-dispersive X-ray spectroscopy
HCM: HyflexCM NiTi instrument
ICP-MS: Inductively coupled plasma mass spectrometry
MS: MiniSCOPE NiTi instrument
NiTi: Nickel-titanium
OC: OneCurve NiTi instrument
PTN: ProTaperNext NiTi instrument
RO: Rotate NiTi instrument
SBF: Simulated body fluid
SEM: Scanning electron microscopy
SS: Stainless-steel hand instrument
